# Gestational age specific stillbirth risk among Indigenous and non-Indigenous women in Queensland, Australia: a population based study

**DOI:** 10.1186/s12884-016-0943-7

**Published:** 2016-07-15

**Authors:** Ibinabo Ibiebele, Michael Coory, Gordon C. S. Smith, Frances M Boyle, Susan Vlack, Philippa Middleton, Yvette Roe, Vicki Flenady

**Affiliations:** Mater Research Institute-University of Queensland (MRI-UQ), Level 2 Aubigny Place, Raymond Terrace, South Brisbane, Queensland 4101 Australia; School of Public Health, University of Queensland, Brisbane, Australia; Murdoch Childrens Research Institute, Melbourne, Australia; Department of Paediatrics, University of Melbourne, Melbourne, Australia; Department of Obstetrics and Gynaecology, University of Cambridge, NIHR Biomedical Research Centre, Cambridge, UK; Queensland Health Metro North Brisbane Public Health Unit, Brisbane, Australia; South Australian Health and Medical Institute & Robinson Research Institute, The University of Adelaide, Adelaide, Australia; Institute for Urban Indigenous Health, Brisbane, Australia

**Keywords:** Aboriginal and Torres Strait Islander Australians, Indigenous, Fetal death, Stillbirth, Risk, Diabetes, Hypertension, Antepartum haemorrhage, Small for gestational age

## Abstract

**Background:**

In Australia, significant disparity persists in stillbirth rates between Aboriginal and Torres Strait Islander (Indigenous Australian) and non-Indigenous women. Diabetes, hypertension, antepartum haemorrhage and small-for-gestational age (SGA) have been identified as important contributors to higher rates among Indigenous women. The objective of this study was to examine gestational age specific risk of stillbirth associated with these conditions among Indigenous and non-Indigenous women.

**Methods:**

Retrospective population-based study of all singleton births of at least 20 weeks gestation or at least 400 grams birthweight in Queensland between July 2005 and December 2011 using data from the Queensland Perinatal Data Collection, which is a routinely-maintained database that collects data on all births in Queensland. Multivariate logistic regression was used to calculate adjusted odds ratios (aOR) and 95 % confidence intervals, adjusting for maternal demographic and pregnancy factors.

**Results:**

Of 360987 births analysed, 20273 (5.6 %) were to Indigenous women and 340714 (94.4 %) were to non-Indigenous women. Stillbirth rates were 7.9 (95 % CI 6.8–9.2) and 4.1 (95 % CI 3.9–4.3) per 1000 births, respectively. For both Indigenous and non-Indigenous women across most gestational age groups, antepartum haemorrhage, SGA, pre-existing diabetes and pre-existing hypertension were associated with increased risk of stillbirth. There were mixed results for pre-eclampsia and eclampsia and a consistently raised risk of stillbirth was not seen for gestational diabetes.

**Conclusion:**

This study highlights gestational age specific stillbirth risk for Indigenous and non-Indigenous women; and disparity in risk at term gestations. Improving access to and utilisation of appropriate and responsive healthcare may help to address disparities in stillbirth risk for Indigenous women.

**Electronic supplementary material:**

The online version of this article (doi:10.1186/s12884-016-0943-7) contains supplementary material, which is available to authorized users.

## Background

Stillbirth rates in Australia have failed to improve over the past two decades. Marked disparity in stillbirth rates persist between Aboriginal and Torres Strait Islander (Indigenous Australian) and non-Indigenous women [1–3]. In 2012, national stillbirth rates among Indigenous women were one and a half times higher (10.8 vs 7.1/1000) than among non-Indigenous women [2]. Moreover, this disparity persists in the rate of term stillbirths (RR 1.71, 95 % CI 1.40-2.09) with little change over time [1].

Diabetes, hypertension, antepartum haemorrhage and small-for-gestational age are important contributors to the higher stillbirth rates observed among Indigenous women [1]. In Queensland, pre-existing and gestational diabetes affected approximately 0.6 and 6.7 % of pregnancies [2]; while Australian national estimates are 0.6 and 4.7 %, respectively [4]. There is evidence of increasing prevalence of pre-existing and gestational diabetes within Queensland [2, 5]; with consistently higher rates of diabetes for Indigenous women compared with non-Indigenous women [4]. However, larger increases in the prevalence of gestational diabetes have been reported for non-Indigenous women [6]. Hypertensive disorders of pregnancy (including pre-existing and pregnancy-induced hypertension) affect around 0.6 and 4.4 % of pregnancies in Queensland [2]. Antepartum haemorrhage (including placenta praevia and abruption) is associated with up to 20 % of very preterm births [7] and affects 2.4 % of pregnancies in Queensland [2]. Indigenous women have higher rates of small-for-gestational age births than non-Indigenous women [8].

Given the contribution of these conditions to stillbirth rates and the disproportionate burden among disadvantaged groups, determining the specific periods of increased risk of stillbirth associated with these conditions is important for clinical management and potential further reductions in stillbirth rates. The objective of this study was to examine the gestational age-specific risk of stillbirth associated with antepartum haemorrhage, hypertension, diabetes and small-for-gestational age among Indigenous and non-Indigenous women in Queensland to determine if there are differential effects of risk factors.

## Methods

We conducted a population-based study utilising data from the Queensland Perinatal Data Collection (QPDC) for the period July 2005 to December 2011. The QPDC is an administrative database which holds data on all births occurring in Queensland. It is a requirement that all births in Queensland are registered in the QPDC for administrative purposes [9].

Demographic factors assessed included maternal Indigenous status, age, marital status, socioeconomic status and geographic location. Indigenous status was based on maternal self-identification as Aboriginal or Torres Strait Islander or not. Relative socioeconomic disadvantage (based on residential postcode) was defined as residing in the lowest ranked 20 % of neighbourhoods. Geographic location (based on residential postcode) was classified as major city, regional or remote. Pregnancy factors assessed included smoking status, substance use, hospital accommodation status, assisted conception use, primigravidity, number of antenatal care visits, gestational age at birth, baby’s sex and small-for-gestational age (SGA). SGA was defined as birthweight less than the 10^th^ Australian population percentile by gestational age, plurality and sex [10].

Medical conditions of interest included: antepartum haemorrhage, essential hypertension, pre-eclampsia/eclampsia, gestational and pre-existing diabetes. Unfortunately, we were unable to assess the effect of overweight/obesity over the study duration. Midwives, who are directly involved in the clinical care of the individual mothers, provide the data to the QPDC. Data audits completed in other Australian states show that false positives and false negatives are generally <5 % for diabetes, hypertensive disorders and antepartum haemorrhage [11]. Stillbirth was defined as fetal death of at least 20 weeks gestation or 400 g birthweight. Stillbirths as a result of terminations of pregnancy for maternal psychosocial reasons, births of unknown maternal Indigenous status or gestational age, births less than 20 weeks and less than 400 g birthweight, and births with a congenital anomaly were excluded.

### Statistical analysis

The all-cause conditional probability of stillbirth occurring at each gestational age interval was calculated using the number of stillbirths occurring within each gestational age interval as the numerator and the number of ongoing pregnancies minus half the number of births occurring within the gestational age interval as the denominator [12].

Risk ratios were used to quantify the unadjusted risk of stillbirth relative to livebirth associated with the conditions of interest (diabetes, hypertension, antepartum haemorrhage and small-for-gestational age). The adjusted stillbirth risk for each of the conditions of interest was assessed in four gestational age intervals of four weeks and an interval of 37 weeks or more using multivariate logistic regression. Regression models were adjusted for the previously listed demographic and pregnancy factors. The denominator was adjusted to reflect the population-at-risk (i.e. the number of ongoing pregnancies at the start of the gestational age interval of interest). Analysis was stratified by maternal Indigenous status, as we hypothesised that differences would be observed in stillbirth risk between Indigenous and non-Indigenous women, furthermore, both groups of women differed significantly on a number of demographic and pregnancy factors. Similar patterns in stillbirth risk were found across strata of antepartum/intrapartum and unknown stillbirth, therefore, to maintain sample size results are presented for all stillbirths combined. Secondary analysis was undertaken on the whole population (Indigenous and non-Indigenous women combined) to assess stillbirth risk among Indigenous women relative to non-Indigenous women. Statistical analyses were performed using Stata/SE for Windows 13.1 (StataCorp LP, College Station, TX, USA 2013). Ethics approval was obtained from the Queensland Health Central Office (Ref: HREC/05/QHC/009), University of Queensland School of Public Health (Ref: II180313) and Mater Health Services (Ref: HREC/15/MHS/36/AM07) Human Research Ethics Committees.

## Results

The characteristics of the study population are summarised in Table 1. A total of 360,987 births were included in the analyses. Of these, 20,273 (5.6 %) births were to Indigenous women and 340,714 (94.4 %) were to non-Indigenous women. The stillbirth rates were 7.9 (95 % CI 6.8–9.2) and 4.1 (95 % CI 3.9–4.3) per 1000 births, respectively; giving a risk ratio of 1.9 (95 % CI 1.6–2.3). For Indigenous and non-Indigenous women, there were higher rates of smoking, substance use, preterm birth and fewer than eight antenatal care visits among women with a stillbirth compared to women with a live birth (Additional file 1: Table S1). Among non-Indigenous women, there were higher rates of socioeconomic disadvantage (risk ratio 1.18, 95 % CI 1.03–1.35) for women with a stillbirth (Additional file 1: Table S1).

**Table 1:** Maternal and pregnancy characteristics by livebirth or stillbirth and Indigenous status

Characteristics	Indigenous (*n* = 20 273)	Non-Indigenous (*n* = 340 714)
Maternal age (years)		
≤ 18 years	1 454 (7.2)	4 402 (1.3)
19–24 years	8 765 (43.2)	67 896 (19.9)
25–30 years	5 693 (28.1)	119 253 (35.0)
31–34 years	2 427 (12.0)	81 322 (23.9)
≥ 35 years	1 934 (9.5)	67 841 (19.9)
Geographic location		
Major City	4 169 (20.6)	209 778 (61.6)
Regional area	11 920 (58.8)	122 590 (36.0)
Remote area	4 184 (20.6)	8 334 (2.5)
Marital status		
Domestic partner	13 033 (64.3)	302 212 (88.7)
No domestic partner	7 232 (35.7)	38 452 (11.3)
Relative socioeconomic disadvantage		
Lowest 20 %	7 422 (36.7)	39 503 (11.6)
Any smoking during pregnancy		
Yes	10 692 (53.1)	56 720 (16.7)
Substance Use during pregnancy		
Yes	343 (1.7)	1 705 (0.5)
Hospital accommodation status		
Public	19 853 (97.9)	229 717 (67.4)
Assisted conception		
Yes	92 (0.5)	12 909 (3.8)
Primigravidity		
Yes	4 834 (23.8)	102 091 (30.0)
Number of antenatal care visits		
Less than 2	1 102 (5.4)	2 291 (0.7)
2 – 4	3 405 (16.8)	17 349 (5.1)
5 – 7	5 828 (28.8)	63 386 (18.6)
8 or more	9 911 (49.0)	257 496 (75.6)

Table 2 shows birth outcomes for the study population. For Indigenous and non-Indigenous women, there were higher rates of pre-existing diabetes, pre-existing hypertension, antepartum haemorrhage and SGA among women with a stillbirth compared to women with a live birth (Table 2). However, there were significant differences in the prevalence of the conditions of interest between Indigenous and non-Indigenous women, respectively as follows: pre-existing diabetes (1.3 % vs 0.5 %, *p* < 0.001), gestational diabetes (6.6 % vs 5.3 %, *p* < 0.001), pre-existing hypertension (1.0 % vs 0.7 %, *p* < 0.001), pre-eclampsia/eclampsia (2.9 % vs 2.2 %, *p* < 0.001), antepartum haemorrhage (2.3 % vs 2.7 %, *p* < 0.001) and small-for-gestational age (15.2 % vs 8.4 %, *p* < 0.001).

**Table 2:** Maternal medical and pregnancy conditions by livebirth or stillbirth and Indigenous status

Characteristics	Indigenous (*n* = 20273)			Non-indigenous (*n* = 340714)		
	Stillbirth (*n* = 160)	Livebirth (*n* = 20113)	Risk Ratio (95 % CI)	Stillbirth (*n* = 1392)	Livebirth (*n* = 339322)	Risk Ratio (95 % CI)
Preterm birth	56 (35.0)	53 (0.3)	132 (94.3–187)	451 (32.4)	243 (0.1)	453 (391–525)
Pre-existing diabetes	12 (7.5)	248 (1.2)	6.08 (3.48–10.6)	21 (1.5)	1 702 (0.5)	3.01 (1.96–4.61)
Gestational diabetes	^a^	1 324 (6.6)	0.38 (0.14–1.00)	58 (4.2)	18 135 (5.3)	0.78 (0.61–1.00)
Pre-existing hypertension	7 (4.4)	190 (0.9)	4.63 (2.21–9.69)	31 (2.2)	2 189 (0.7)	3.45 (2.43–4.90)
Pre-eclampsia/Eclampsia	^a^	574 (2.9)	1.10 (0.46–2.60)	77 (5.5)	7 359 (2.2)	2.55 (2.05–3.17)
Pregnancy induced hypertension	^a^	488 (2.4)	0.26 (0.04–1.82)	27 (1.9)	9 897 (2.9)	0.67 (0.46–0.97)
Antepartum haemorrhage	44 (27.5)	412 (2.0)	13.4 (10.3–17.6)	337 (24.2)	8 948 (2.6)	9.18 (8.35–10.1)
Small-for-gestational age	58 (36.3)	3 021 (15.0)	2.46 (2.00–3.02)	410 (29.5)	28 214 (8.3)	3.58 (3.30–3.89)

^a^Numbers and percentages not displayed for cells with five or less observations to protect participant privacy

ICD10-AM codes: Antepartum haemorrhage (O44.1, O45-O46), Pre-existing hypertension (O10.0, O10.2-10.4, O10.9, O11), Pregnancy Induced hypertension (O13), Pre-eclampsia/Eclampsia (O14, O15), Pre-existing diabetes (O24.0, O24.1, O24.3, O24.8), Gestational diabetes (O24.4, O24.9)

Small-for-gestational age = birthweight less than the 10^th^ Australian population percentile for gestational age, plurality and sex

**Fig. 1:**
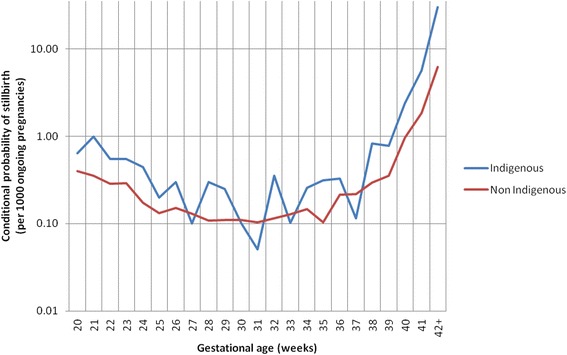
Gestational age specific risk of stillbirth by Indigenous status. Numerator = the number of stillbirths occurring at each week of gestational age. Denominator = the number of ongoing pregnancies at each gestational age week minus half the number of births occurring within the gestational age week

The all-cause stillbirth risk profile for Indigenous and non-Indigenous women was characterised by lower rates of stillbirth at gestational ages before 38 weeks (0.2 to 0.6/1000 and 0.1 to 0.3/1000 ongoing pregnancies for Indigenous and non-Indigenous women, respectively). A marked increase in risk was then observed from 39 weeks onwards, with a two-fold or higher risk for Indigenous women compared with non-Indigenous women (Fig. 1).

Overall, women with a stillbirth were more likely to have: pre-existing diabetes (2.1 % vs 0.5 %, risk ratio 3.92, 95 % CI 2.79–5.51), pre-existing hypertension (2.5 % vs 0.7 %, risk ratio 3.70, 95 % CI 2.70–5.08), pre-eclampsia/eclampsia (5.3 % vs 2.2 %, risk ratio 2.39, 95 % CI 1.94–2.96), antepartum haemorrhage (24.6 % vs 2.6 %, risk ratio 9.43, 95 % CI 8.62–10.3) and a small for gestational age infant (30.5 % vs 8.7 %, risk ratio 3.47, 95 % CI 3.21–3.75), while women with gestational diabetes (4.0 % vs 5.4 %, risk ratio 0.74, 95 % CI 0.58–0.94) or pregnancy-induced hypertension (1.8 % vs 2.9 %, risk ratio 0.62, 95 % CI 0.43–0.90) were less likely to have a stillbirth.

Gestational age-specific odds of stillbirth are presented by condition for Indigenous and non-Indigenous women separately in Table 3; and for the whole population in Additional file 1: Table S2. Additional file 1: Table S3 shows the odds of stillbirth by condition of interest for Indigenous women relative to non-Indigenous women.

**Table 3:** Gestational age-specific risk of stillbirth by medical condition and Indigenous status

Conditions	Indigenous (*n* = 20,273 births)				
	Adjusted Odds Ratios (95 % Confidence Intervals)				
	20–23 weeks	24–27 weeks	28–32 weeks	33–36 weeks	37–42+ weeks
Pre-existing diabetes^a,c^	1.42 (0.17–11.6)	3.15 (0.31–31.7)	7.81 (1.28–47.8)	19.0 (5.30–68.4)	15.4 (4.79–49.2)
Gestational diabetes^a,c^	-	-	-	0.68 (0.09–5.32)	0.93 (0.22–4.01)
Pre-existing hypertension ^b,c^	2.27 (0.27–18.8)	5.62 (0.55–57.7)	13.2 (2.07–84.5)	1.35 (0.14–12.8)	1.49 (0.15–14.8)
Pre-Eclampsia/Eclampsia ^c^	0.70 (0.09–5.14)	1.59 (0.21–12.2)	-	1.92 (0.24–15.2)	3.32 (0.79–14.0)
Antepartum haemorrhage^a^	18.1 (9.91–33.1)	14.2 (5.14–39.4)	31.2 (11.9–81.6)	2.53 (0.31–20.3)	17.1 (7.28–40.0)
Small-for-gestational age	1.30 (0.67–2.52)	3.32 (1.31–8.44)	7.04 (2.70–18.4)	3.05 (1.16–8.02)	2.10 (1.08–4.08)
Conditions	Non-Indigenous (*n* = 340,714 births)				
	Adjusted Odds Ratios (95 % Confidence Intervals)				
	20–23 weeks	24–27 weeks	28–32 weeks	33–36 weeks	37–42+ weeks
Pre-existing diabetes^a^	0.90 (0.21–3.94)	3.23 (0.98–10.7)	1.33 (0.18–9.64)	7.68 (3.36–17.6)	6.77 (3.47–13.2)
Gestational diabetes^a^	0.47 (0.21–1.06)	0.76 (0.31–1.87)	1.25 (0.61–2.56)	1.94 (1.14–3.32)	1.29 (0.84–1.97)
Pre-existing hypertension ^b^	7.27 (4.18–12.6)	6.59 (2.99–14.6)	3.35 (1.05–10.7)	2.54 (0.80–8.11)	1.29 (0.41–4.04)
Pre-eclampsia/Eclampsia	0.99 (0.51–1.94)	6.40 (4.14–9.91)	5.55 (3.43–8.99)	2.00 (0.94–4.26)	2.77 (1.65–4.66)
Antepartum haemorrhage^a^	9.03 (7.21–11.3)	7.99 (5.67–11.3)	12.8 (9.25–17.9)	17.9 (13.1–24.4)	7.95 (5.85–10.8)
Small-for-gestational age	3.73 (3.00–4.64)	6.43 (4.78–8.67)	3.85 (2.77–5.33)	3.49 (2.50–4.87)	3.72 (2.91–4.74)

Regression models adjusted for maternal age, smoking status, remoteness, substance use, gender, gravidity, hospital accommodation status, assisted conception use, socioeconomic status, marital status, number of antenatal care visits

^a^Models additionally adjusted for pre-existing hypertension

^b^These models additionally adjusted for pre-existing diabetes

^c^Exact logistic regression model

### Diabetes

There were significantly increased odds of stillbirth associated with pre-existing diabetes from 33 weeks onwards for both groups of women. Among Indigenous women, the odds of stillbirth at 33–36 and 37+ weeks were aOR 19.0 (95 % CI 5.3–68) and aOR 15.4 (95 % CI 4.8–49), respectively. Among non-Indigenous women, the adjusted odds ratios for the equivalent gestational ages were 7.7 (95 % CI 3.4–17.6) and 6.8 (95 % CI 3.5–13.2) (Table 3). There was a suggestion of increased odds of stillbirth associated with gestational diabetes from 33 weeks onwards among non-Indigenous women. There were decreased odds of stillbirth associated with diabetes at gestational ages less than 28 weeks for Indigenous women, but increased odds at term (Additional file 1: Table S3).

### Hypertension

There was a suggestion of increased odds of stillbirth associated with pre-existing hypertension at all gestational ages assessed. Pre-eclampsia/eclampsia was likely to be associated with increased odds of stillbirth for both groups of women, although numbers were too small in some instances to reach conventional statistical significance of 0.05. As with diabetes, there were decreased odds of stillbirth associated with hypertensive disorders at gestational ages less than 28 weeks for Indigenous women, and increased odds at term (Additional file 1: Table S3).

### Antepartum haemorrhage

Antepartum haemorrhage was strongly associated with stillbirth for most gestational age groups and for both Indigenous and non-Indigenous women. Among non-Indigenous women, odds of stillbirth for antepartum haemorrhage was higher at 33–36 weeks (aOR 17.9, 95 % CI 13.1–24.4) compared to odds at less than 28 weeks or at term (Table 3). Compared to non-Indigenous women, there were decreased odds of stillbirth at 24–27 weeks (aOR 0.56, 95 % CI 0.33–0.96) but increased odds of stillbirth at 37+ weeks (aOR 1.53, 95 % CI 1.07–2.21) (Additional file 1: Table S3).

### Small-for-gestational age (SGA)

There were significantly increased odds of stillbirth associated with SGA from 24 weeks gestation onwards for Indigenous women; and at all gestational age groups for non-Indigenous women (Table 3). Indigenous women had decreased odds of stillbirth associated with SGA at gestational ages less than 28 weeks compared with non-Indigenous women, but there was no difference in stillbirth risk at 28 weeks or older (Additional file 1: Table S3).

## Discussion

### Main findings

This study found increased odds of stillbirth associated with pre-existing diabetes, pre-existing hypertension, antepartum haemorrhage and SGA across most gestational age groups for both Indigenous and non-Indigenous women after adjusting for potential confounders. There were mixed results for pre-eclampsia/eclampsia and gestational diabetes. At less than 27 weeks, there were decreased odds of stillbirth associated with diabetes, pre-existing hypertension and SGA for Indigenous compared with non-Indigenous women. Conversely, the odds of stillbirth for diabetes, hypertension and antepartum haemorrhage were 1 ½ times higher at term for Indigenous women as non-Indigenous women. The protective effect observed at lower gestational ages may be due to detection bias or differing causes of stillbirth; higher rates of spontaneous preterm birth have been reported for Indigenous women [13]. Likewise, the increased odds of stillbirth may reflect the impact of lower levels of antenatal care such as decreased detection of SGA and suboptimal diabetes management. Few studies have assessed gestational age-specific stillbirth risk using the population-at-risk approach within this study population [14]. The profile of all-cause stillbirth risk for both groups of women was similar to profiles reported in populations in USA [15]. However, the overall magnitude of risk for Indigenous women was about twice that of non-Indigenous women.

We acknowledge that the study was underpowered to detect interactions between Indigenous status and stillbirth risk factors which may have indicated differential effects of risk factors within the two groups of women.

### Diabetes

We found increased odds of stillbirth associated with pre-existing diabetes from 33 weeks onwards for both groups of women. Similar findings of increased risk of stillbirth due to pre-existing diabetes from 32 weeks onwards have been reported elsewhere [16, 17]. The one and a half fold disparity in the magnitude of stillbirth risk between Indigenous and non-Indigenous women at term mirrors higher rates of pre-existing diabetes among Indigenous women in our study (1.3 % versus 0.5 %). Similarly, our finding of increased odds of stillbirth due to gestational diabetes from 28 weeks onwards among non-Indigenous women concurs with Hutcheon and colleagues [18]. While the prevalence of gestational diabetes have been reported to be higher among Indigenous women, the rate of increase of gestational diabetes over time was found to be greater among non-Indigenous women [4].

Current management for pre-existing diabetes includes strict glycaemic control, pre-conceptual folate supplementation, cessation of oral hypoglycaemic agents, diabetes complication review, periodic ultrasound scans for fetal morphology (18–20 weeks), cardiac views (24 weeks), fetal growth (28–30 and 34–36 weeks) [19]. The International Association of Diabetes and Pregnancy Study Group recommend screening all women at their first antenatal visit for gestational diabetes or previously undiagnosed pre-existing diabetes [20]; however, a tiered approach with early screening of women at high risk or with multiple risk factors is recommended in Australia [21]. Perinatal mortality audits in high income countries have identified poor glycaemic control [22, 23] and inadequate screening among women at risk [24] as contributing to stillbirth. Conversely, pre-pregnancy counselling has been found to significantly lower the risk of major congenital anomalies associated with diabetes during pregnancy (RR 0.36, 95 % CI 0.22–0.59; absolute risk 2.1 % versus 6.5 %) [21]. Pre-conception care was found to reduce perinatal mortality while optimal vs suboptimal serum blood glucose control was associated with reduced perinatal mortality (RR 0.40, 95 % CI 0.25–0.63) but not stillbirth (RR 0.51 95 % CI 0.14–1.88) [25]. Our findings highlight the need for early initiation of monitoring of women with pre-existing diabetes, especially for Indigenous women; and early identification and management of gestational diabetes.

### Antepartum haemorrhage

We found increased risk of stillbirth associated with antepartum haemorrhage at all gestational age groups assessed; and the magnitude of risk for Indigenous women was 1 ½ times higher than for non-Indigenous women at term. Despite differences in methodology, similar magnitude of risk has been reported in population based studies from Canada and USA with adjusted odds ratios ranging from 11.40-18.90 for stillbirth associated with placental abruption in births of at least 20 weeks [26, 27]. The disparity in risk seen between Indigenous and non-Indigenous women may be a reflection of higher prevalence of risk factors for placenta praevia and abruption such as maternal smoking (52.7 % vs 16.6 %), substance use (1.7 % vs 0.5 %) and small for gestational age (15.2 % vs 8.4 %). At present, there is limited evidence for the prediction or prevention of abruption; and antepartum haemorrhage usually constitutes a sudden obstetric emergency. The mainstay of management for antepartum haemorrhage includes: assessment of maternal and fetal condition, prompt resuscitation if required and early delivery if there is fetal distress or the baby is suitably mature [28, 29]. It is estimated that up to 70 % of antepartum haemorrhage cases occur in apparently low risk pregnancies [30].

### Small-for-gestational age (SGA)

We found increased odds of stillbirth associated with SGA from 24 weeks onwards for both Indigenous and non-Indigenous women; and similar findings have been reported elsewhere [16]. Although not reflected in the stillbirth odds, there was a higher prevalence of SGA among Indigenous women in this study (15.2 % vs 8.4 %), similar to reports from the Northern Territory (Indigenous 11.9 % vs non-Indigenous 5.0 %) [31].

SGA has been used as a proxy for fetal growth restriction and undetected fetal growth restriction has been identified as a significant potentially modifiable risk factor for stillbirth [32]. There are currently no antenatal interventions to treat fetal growth restriction and the mainstay of management is fetal monitoring to determine the optimal timing for delivery (balancing risks and benefits of adverse fetal outcomes against morbidity and mortality associated with early delivery at a given gestational age) [33]. Management of SGA involves accurate determination of gestational age and serial monitoring of fetal growth (using symphysis-fundal height measurement or ultrasound biometry). However, controversy exists over the accuracy of symphysis-fundal height measurement especially in obese women [34] and the use of customised growth charts due to lack of high level evidence [35]. A number of interventions have been found to be effective in the prevention of SGA among women at increased risk, including: antiplatelet agents, smoking cessation, progesterone therapy, anti-thrombotic therapy and interventionist care in severe pre-eclampsia [36]. Further studies are needed into preventive strategies for SGA that also include pre-eclampsia and preterm birth [36], as well as investigating the serious adverse effects of antenatal antithrombotic therapies [37].

### Equity in access to antenatal care

Overall, this study highlights the importance of optimal maternal health prior to pregnancy as well as early initiation of high quality antenatal care in the context of continued disparity in risk of stillbirth among Indigenous and non-Indigenous women. Equity in access to antenatal care services and their utilisation is important to addressing disparities in health outcomes for all women, particularly Indigenous women [38]. Indigenous women in our study were more likely to have fewer antenatal care visits, a finding supported by others [2, 13]. Limited availability of culturally appropriate services may affect attendance for antenatal care; for example there were on average 5.5 antenatal care visits within mainstream services versus 10.5 visits within community controlled service settings for Indigenous women [39]. Active efforts to ensure appropriate and responsive care in the clinical environment both at the level of the individual health practitioner and within mainstream health care services are essential to reduce or eliminate social barriers to accessing health care. Embedding cultural competence in continuing organisational quality improvement processes has been shown to enhance health outcomes for Indigenous people [40]. This is especially important for Indigenous women who seek care within mainstream health services.

The Australian government has recognised the importance of maternal and child health to the “Closing the Gap” initiative and has prioritised maternal and child health [41]. It has been shown that an investment in stillbirth prevention provides a three-fold return in terms of maternal, neonatal and child health [42]. While there has been continued support for Aboriginal and Torres Strait Islander community controlled health centres and their Mums and Bubs centres, many health and lifestyle modification programs have been defunded. Declines seen in smoking and under-5 child mortality rates are further evidence for the need to maintain funding for successful lifestyle modification programs and antenatal care services operated by community controlled health services [41]. More broadly, access to primary care services is critical to reducing health inequity and policies that undermine universal health care pose a significant threat to this goal by presenting further financial barriers that are likely to disproportionately affect the most vulnerable [43].

## Conclusions

This study highlights the gestational age specific stillbirth risk associated with diabetes, hypertension, antepartum haemorrhage and small for gestational age. It also highlights the disparity in stillbirth risk between Indigenous and non-Indigenous women at term and the need to prioritise early detection and management of these conditions and to work with women before, during and between pregnancies. Improving access to and utilisation of appropriate and responsive healthcare may help to address disparities in stillbirth risk for Indigenous women. Larger population-based studies are needed to re-evaluate whether there are differences in the effect of risk factors on stillbirth risk among Indigenous and non-Indigenous women.

## Abbreviations

aOR, adjusted odds ratio; QPDC, Queensland Perinatal Data Collection; SGA, small for gestational age

## Additional file

Additional file 1: Table S1. Maternal and pregnancy characteristics by livebirth or stillbirth and Indigenous status. **Table S2**. Gestational age-specific risk of stillbirth associated with diabetes, hypertension, antepartum haemorrhage and SGA, combined Indigenous and non-Indigenous women, Queensland, mid 2005–2011. **Table S3**. Effect of Indigenous status on gestational age specific stillbirth risk (Indigenous relative to non-Indigenous). (DOCX 17 kb)

## References

[1] Ibiebele I (2015). Stillbirth rates among Indigenous and non-Indigenous women in Queensland, Australia: is the gap closing?. BJOG.

[2] Hilder L (2014). Australia’s mothers and babies 2012. Perinatal statistics series no. 30. Cat. no. PER 69.

[3] Flenady V (2016). Stillbirths: recall to action in high-income countries. Lancet.

[4] Australian Institute of Health and Welfare (2010). Diabetes in pregnancy: its impact on Australian women and their babies. Diabetes series no. 14. Cat. no. CVD 52.

[5] Li Z (2011). Australia’s mothers and babies 2009. Perinatal Statistics Series No. 25. Cat. no. PER 52.

[6] Chamberlain C (2014). Prevalence of gestational diabetes mellitus among Indigenous women and comparison with non-Indigenous Australian women: 1990–2009. Aust N Z J Obstet Gynaecol.

[7] Hagan R (1996). Very preterm birth - a regional study. Part 1: Maternal and obstetric factors. Br J Obstet Gynaecol.

[8] Panaretto K (2006). Risk factors for preterm, low birth weight and small for gestational age birth in urban Aboriginal and Torres Strait Islander women in Townsville. Aust N Z J Public Health.

[9] Queensland Health (2013). Perinatal Statistics Queensland 2011.

[10] Dobbins TA (2012). Australian national birthweight percentiles by sex and gestational age, 1998–2007. Med J Aust.

[11] Metcalfe A (2012). Maternal morbidity data in Australia: an assessment of the feasibility of standardised collection. Cat no. PER.

[12] Smith GC (2001). Life-table analysis of the risk of perinatal death at term and post term in singleton pregnancies. Am J Obstet Gynecol.

[13] Leeds K (2007). Indigenous mothers and their babies, Australia 2001–2004. AIHW cat. no. PER 38. Perinatal Statistics Series no. 19.

[14] Coory M (1998). Gestational-age-specific stillbirth risk among Australian Aborigines. Int J Epidemiol.

[15] Heuser C (2010). Non-anomalous stillbirth by gestational age: Trends differ based on method of epidemiologic calculation. J Matern Fetal Neonatal Med.

[16] Canterino JC (2004). Maternal age and risk of fetal death in singleton gestations: USA, 1995–2000. J Matern Fetal Neonatal Med.

[17] Holman N (2014). Women with pre-gestational diabetes have a higher risk of stillbirth at all gestations after 32 weeks. Diabet Med.

[18] Hutcheon JA (2013). Immortal Time Bias in the Study of Stillbirth Risk Factors: The Example of Gestational Diabetes. Epidemiology.

[19] McElduff A (2005). The Australasian Diabetes in Pregnancy Society consensus guidelines for the management of type 1 and type 2 diabetes in relation to pregnancy. Med J Aust.

[20] American Diabetes Association (2015). 12. Management of Diabetes in Pregnancy. Diabetes Care.

[21] Nankervis A (2013). Australasian Diabetes In Pregnancy Society (ADIPS) Consensus Guidelines for the Testing and Diagnosis of Gestational Diabetes Mellitus in Australia.

[22] Wolleswinkel-van den Bosch JH (2002). Substandard factors in perinatal care in The Netherlands: a regional audit of perinatal deaths. Acta Obstet Gynecol Scand.

[23] Lauenborg J (2003). Audit on stillbirths in women with pregestational type 1 diabetes. Diabetes Care.

[24] Saastad E, Vangen S, Frøen JF (2007). Suboptimal care in stillbirths - a retrospective audit study. Acta Obstet Gynecol Scand.

[25] Syed M (2011). Effect of screening and management of diabetes during pregnancy on stillbirths. BMC Public Health.

[26] Salihu HM (2005). Perinatal mortality associated with abruptio placenta in singletons and multiples. Am J Obstet Gynecol.

[27] McDonald SD, Vermeulen MJ, Ray JG (2007). Risk of fetal death associated with maternal drug dependence and placental abruption: a population-based study. J Obstet Gynaecol Can.

[28] Neilson JP (2003). Interventions for treating placental abruption. Cochrane Database Syst Rev.

[29] Royal College of Obstetricians and Gynaecologists (2011). Antepartum Haemorrhage (Green-top Guideline No. 63).

[30] Toivonen S (2002). Reproductive risk factors, Doppler findings, and outcome of affected births in placental abruption: a population-based analysis. Am J Perinatol.

[31] Kalro A, Singh G (2014). Big things come from small beginnings: an audit of prevalence of fetal growth restriction and its causes in the Northern Territory. J Paediatr Child Health.

[32] Gardosi J et al. Maternal and fetal risk factors for stillbirth: population based study. BMJ. 2013;346. 10.1136/bmj.f108 PMC3554866 23349424

[33] Alberry M, Soothill P (2007). Management of fetal growth restriction. Arch Dis Child Fetal Neonatal Ed.

[34] Jelks A, Cifuentes R, Ross M (2007). Clinician bias in fundal height measurement. Obstet Gynecol.

[35] Carberry A (2011). Customised versus population-based growth charts as a screening tool for detecting small for gestational age infants in low-risk pregnant women. Cochrane Database Syst Rev.

[36] Morris RK (2013). Effectiveness of interventions for the prevention of small-for-gestational age fetuses and perinatal mortality: a review of systematic reviews. Acta Obstet Gynecol Scand.

[37] Dodd JM et al. Antithrombotic therapy for improving maternal or infant health outcomes in women considered at risk of placental dysfunction. Cochrane Database Syst Rev., 2013(7): p. Art. No.: CD006780 10.1002/14651858.CD006780.pub3 PMC11365097 23884904

[38] Rumbold A (2011). Delivery of maternal health care in Indigenous primary care services: baseline data for an ongoing quality improvement initiative. BMC Pregnancy Childbirth.

[39] Rumbold AR, Cunningham J (2008). A review of the impact of antenatal care for Australian Indigenous women and attempts to strengthen these services. Matern Child Health J.

[40] Reibel T, Walker R (2010). Antenatal services for Aboriginal women: the relevance of cultural competence. Qual Prim Care.

[41] Holland C. Close the Gap - progress and priorities report 2014. Australian Human Rights Commission; 2014. https://www.humanrights.gov.au/sites/default/files/document/publication/ctg-progress-and-priorities-report.pdf

[42] Bhutta ZA (2011). Stillbirths: what difference can we make and at what cost?. Lancet.

[43] Starfield B, Shi L, Macinko J (2005). Contribution of Primary Care to Health Systems and Health. Milbank Q.

