# Supplementary material for: Gestational age specific stillbirth risk among Indigenous and non-Indigenous women in Queensland, Australia: a population based study
Source: BMC Pregnancy Childbirth. 2016 Jul 15;16:159. doi: 10.1186/s12884-016-0943-7 (PMC4946098; doi:10.1186/s12884-016-0943-7)
Supplement: Additional file 1: Table S1. — Maternal and pregnancy characteristics by livebirth or stillbirth and Indigenous status. Table S2. Gestational age-specific risk of stillbirth associated with diabetes, hypertension, antepartum haemorrhage and SGA, combined Indigenous and non-Indigenous women, Queensland, mid 2005–2011. Table S3. Effect of Indigenous status on gestational age specific stillbirth risk (Indigenous relative to non-Indigenous). (DOCX 17 kb) [file 12884_2016_943_MOESM1_ESM.docx]

**Additional file 1: Table S1: Maternal and pregnancy characteristics by livebirth or stillbirth and Indigenous status**

| **Characteristics** | **Indigenous (n=20273)** | | | **Non-Indigenous (n=340714)** | | |
| --- | --- | --- | --- | --- | --- | --- |
|  | **Stillbirth (n=160)** | **Livebirth**  **(n=20113)** | **Risk Ratio**  **(95% CI)** | **Stillbirth (n=1392)** | **Livebirth (n=339322)** | **Risk Ratio**  **(95% CI)** |
| **Maternal age (years)** |  |  |  |  |  |  |
| ≤18 years | 10 (6.3) | 1 444 (7.2) | 0.87 (0.48-1.59) | 42 (3.0) | 4 360 (1.3) | 2.35 (1.74-3.17) |
| 19-24 years | 67 (41.9) | 8 698 (43.3) | 0.97 (0.81-1.16) | 313 (22.5) | 67 583 (19.9) | 1.13 (1.02-1.24) |
| 25-30 years | 48 (30.0) | 5 645 (28.1) | 1.07 (0.84-1.36) | 437 (31.4) | 118 816 (35.0) | 0.90 (0.83-0.97) |
| 31-34 years | 14 (8.8) | 2 413 (12.0) | 0.73 (0.44-1.20) | 290 (20.8) | 81 032 (23.9) | 0.87 (0.79-0.97) |
| ≥35 years | 21 (13.1) | 1 913 (9.5) | 1.38 (0.92-2.06) | 310 (22.3) | 67 531 (19.9) | 1.12 (1.01-1.23) |
| **Geographic Location** |  |  |  |  |  |  |
| Major City | 26 (16.3) | 4 143 (20.6) | 0.79 (0.55-1.12) | 835 (60.0) | 208 943 (61.6) | 0.97 (0.93-1.02) |
| Regional area | 95 (59.4) | 11 825 (58.8) | 1.01 (0.89-1.15) | 525 (37.7) | 122 065 (36.0) | 1.05 (0.98-1.12) |
| Remote area | 39 (24.4) | 4 145 (20.6) | 1.18 (0.90-1.56) | 31 (2.2) | 8 303 (2.5) | 0.91 (0.64-1.29) |
| **Marital Status** |  |  |  |  |  |  |
| Domestic partner | 102 (63.8) | 12 931 (64.3) | 0.99 (0.88-1.11) | 1 148 (82.5) | 301 064 (88.7) | 0.93 (0.91-0.95) |
| No domestic partner | 58 (36.3) | 7 174 (35.7) | 1.02 (0.83-1.25) | 241 (17.3) | 38 211 (11.3) | 1.54 (1.37-1.73) |
| **Relative socioeconomic disadvantage** | | | | | | |
| Lowest 20% | 59 (36.9) | 7 363 (36.6) | 1.01 (0.82-1.23) | 190 (13.6) | 39 313 (11.6) | 1.18 (1.03-1.35) |
| **Any smoking during pregnancy** | | | | | | |
| Yes | 95 (59.4) | 10 597 (52.7) | 1.17 (1.03-1.33) | 332 (23.9) | 56 388 (16.6) | 1.47 (1.34-1.62) |
| **Substance Use during pregnancy** | | | | | | |
| Yes | 11 (6.9) | 332 (1.7) | 4.17 (2.33-7.44) | 15 (1.1) | 1 690 (0.5) | 2.16 (1.31-3.59) |
| **Hospital accommodation status** | | | | | | |
| Public | 157 (98.1) | 19 696 (97.9) | 1.00 (0.98-1.02) | 1 084 (77.9) | 228 633 (67.4) | 1.16 (1.13-1.19) |
| **Assisted Conception** |  |  |  |  |  |  |
| Yes | ^ | 91 (0.5) | 1.38 (0.19-9.85) | 66 (4.7) | 12 843 (3.8) | 1.26 (0.99-1.59) |
| **Primiparity** |  |  |  |  |  |  |
| Yes | 37 (23.1) | 4 797 (23.9) | 0.97 (0.73-1.29) | 418 (30.0) | 101 673 (30.0) | 1.00 (0.93-1.09) |
| **Number of antenatal care visits** | | | | | | |
| Less than 2 | 47 (29.4) | 1 055 (5.2) | 5.66 (4.43-7.25) | 147 (10.6) | 2 144 (0.6) | 16.8 (14.4-19.7) |
| 2 – 4 | 57 (35.6) | 3 348 (16.6) | 2.16 (1.75-2.67) | 459 (33.0) | 16 890 (5.0) | 6.68 (6.19-7.21) |
| 5 – 7 | 23 (14.4) | 5 805 (28.9) | 0.50 (0.35-0.74) | 367 (26.4) | 63 019 (18.6) | 1.43 (1.31-1.56) |
| 8 or more | 31 (19.4) | 9 880 (49.1) | 0.40 (0.29-0.55) | 407 (29.2) | 257 089 (75.8) | 0.39 (0.36-0.42) |

^Numbers and percentages not displayed for cells with 5 observations or less

**Table S2: Gestational age-specific risk of stillbirth associated with diabetes, hypertension, antepartum haemorrhage and SGA, combined Indigenous and non-Indigenous women, Queensland, mid 2005-2011**

| **Conditions** | **All births (n=360 987)** | | | | |
| --- | --- | --- | --- | --- | --- |
|  | **Adjusted Odds Ratios (95% Confidence Intervals)** | | | | |
|  | **20-23 weeks** | **24-27 weeks** | **28-32 weeks** | **33-36 weeks** | **37-42+ weeks** |
| Pre-existing diabetes^a^ | 1.02 (0.31-3.37) | 3.34 (1.17-9.57) | 3.00 (0.92-9.78) | 7.28 (3.35-15.8) | 8.26 (4.70-14.5) |
| Gestational diabetes^a^ | 0.41 (0.18-0.91) | 0.68 (0.28-1.67) | 1.12 (0.55-2.28) | 1.80 (1.07-3.02) | 1.24 (0.82-1.87) |
| Pre-existing hypertension ^b^ | 6.30 (3.70-10.7) | 6.29 (2.97-13.3) | 4.39 (1.75-11.0) | 2.76 (1.00-7.64) | 1.36 (0.50-3.70) |
| Pre-Eclampsia/Eclampsia | 0.92 (0.49-1.73) | 5.50 (3.57-8.46) | 4.47 (2.76-7.26) | 2.10 (1.03-4.27) | 2.63 (1.61-4.30) |
| Antepartum haemorrhage^a^ | 9.68 (7.85-12.0) | 8.49 (6.14-11.7) | 13.8 (10.1-18.6) | 16.4 (12.1-22.2) | 8.44 (6.32-11.3) |
| Small-for-gestational age | 3.26 (2.64-4.02) | 5.95 (4.47-7.93) | 3.98 (2.93-5.40) | 3.51 (2.56-4.81) | 3.35 (2.66-4.22) |

Regression models adjusted for maternal age, smoking status, remoteness, substance use, gender, parity, hospital accommodation status, assisted conception use, socioeconomic status, marital status, number of antenatal care visits.

^a^Models additionally adjusted for pre-existing hypertension.

^b^ These models additionally adjusted for pre-existing diabetes.

**Table S3: Effect of Indigenous status on gestational age specific stillbirth risk (Indigenous relative to non-Indigenous)**

| **Conditions** | **All births (n=360 987)** | | | | |
| --- | --- | --- | --- | --- | --- |
|  | **Adjusted Odds Ratios (95% Confidence Intervals)** | | | | |
|  | **20-23 weeks** | **24-27 weeks** | **28-32 weeks** | **33-36 weeks** | **37-42+ weeks** |
| Pre-existing diabetes^a^ | **0.71 (0.51-0.98)** | **0.51 (0.30-0.86)** | 0.66 (0.39-1.11) | 0.86 (0.51-1.46) | **1.45 (1.01-2.09)** |
| Gestational diabetes^a^ | **0.71 (0.51-0.99)** | **0.53 (0.31-0.89)** | 0.67 (0.40-1.12) | 0.89 (0.52-1.51) | **1.49 (1.04-2.15)** |
| Pre-existing hypertension ^b^ | **0.71 (0.51-0.98)** | **0.51 (0.30-0.86)** | 0.66 (0.39-1.11) | 0.86 (0.51-1.46) | **1.45 (1.01-2.09)** |
| Pre-Eclampsia/Eclampsia | 0.72 (0.52-1.00) | **0.53 (0.31-0.89)** | 0.67 (0.40-1.12) | 0.90 (0.53-1.53) | **1.50 (1.04-2.15)** |
| Antepartum haemorrhage^a^ | 0.77 (0.55-1.08) | **0.56 (0.33-0.96)** | 0.73 (0.43-1.23) | 0.99 (0.58-1.69) | **1.53 (1.07-2.21)** |
| Small-for-gestational age | **0.68 (0.49-0.95)** | **0.49 (0.29-0.83)** | 0.58 (0.34-0.99) | 0.85 (0.50-1.45) | 1.42 (0.98-2.04) |

Regression models adjusted for maternal age, smoking status, remoteness, substance use, gender, parity, hospital accommodation status, assisted conception use, socioeconomic status, marital status, number of antenatal care visits.

^a^Models additionally adjusted for pre-existing hypertension.

^b^ These models additionally adjusted for pre-existing diabetes.
